# Rill erosion dynamics in smallholder farming systems of wet tropical Africa

**DOI:** 10.1038/s41598-026-50821-7

**Published:** 2026-05-21

**Authors:** Florian Wilken, Peter Fiener, Pedro Batista, Matthew Cooper, Jasmine Haist, Daniel Muhindo, Kristof van Oost, Martin Rueegg, Sebastian Doetterl

**Affiliations:** 1 Institute for Geography, Universität Augsburg, Augsburg, Germany; 2 Department of Environmental Systems Science, Eidgenössische Technische Hochschule Zürich, Zurich, Switzerland; 3 Faculty of Agronomy, Université Catholique de Bukavu, Bukavu, Democratic Republic of Congo; 4 Earth and Life Institute, Université Catholique de Louvain, Louvain-la-Neuve, Belgium

**Keywords:** Event frequency, High revisit rate monitoring, Intra-field connectivity, Rill erosion, UAV aerial photographs, Ecology, Ecology, Environmental sciences, Natural hazards

## Abstract

Tropical Africa is globally one of the most sensitive regions to accelerated soil erosion, and much of its cropland is characterised by a substantial yield gap. In particular, the White Nile-Congo ridge (NiCo) region of the eastern Democratic Republic of the Congo (DR Congo) and Uganda is a hotspot for issues relating to food security driven by soil degradation due to steep terrain, highly erosive rainfall and low soil cover. Most soil erosion studies in the region are based on plot or large-scale modelling. Both approaches lack information on inter-field connectivity processes, which are especially important in smallholder farming systems with average field sizes below 0.1 ha. To address this knowledge gap, an unmanned aerial vehicle (UAV) based high-spatial and temporal-resolution monitoring campaign was carried out over four smallholder farming areas within the eastern DR Congo and western Uganda, with substantial differences in cropland management and productivity. The campaign covered 833 individual fields, which were monitored up to twice per month (for two years) using UAV-based aerial photography to provide insight into event-based rill erosion processes and landscape connectivity. The aerial photography data were classified according to field conditions: (i) dense vegetation cover, (ii) low vegetation cover or bare soil without signs of rill erosion, (iii) low vegetation cover or bare soil with signs of rill erosion. Land-use patchiness associated with smallholder farming systems was found to reduce inter-field connectivity and to promote highly localised rill development. Furthermore, rill erosion in the NiCo region is not an episodic process but takes place regularly during the rainy season due to frequent storm events falling on bare soil in fields left fallow for individual cultivation periods. Soil erosion dynamics of smallholder farming regions in the study area, therefore, pose unresolved challenges regarding their implementation in large-scale predictions.

**Supplementary Information:**

The online version contains supplementary material available at 10.1038/s41598-026-50821-7.

## Introduction

Smallholder farming, with farm sizes below 2 ha, feeds a large portion of the global population^1–3^. In sub-Saharan Africa, 80% of all farms fall in the category of smallholder farming, comprising between 30% and 40% of total agricultural land^3^. Smallholder farming in sub-Saharan Africa is often associated with low nutrient input management^4,5^ leading to substantial yield gaps^6,7^. Contrary to the global trend of improving food security and increasing farm sizes, (sub-Saharan) Africa shows decreasing per-capita yields^6^ and decreasing average farm sizes^3^ 1960: 2.9 ha – 2010: 1.6 ha, which is indicative of stagnating agricultural developments.

**Fig. 1:**
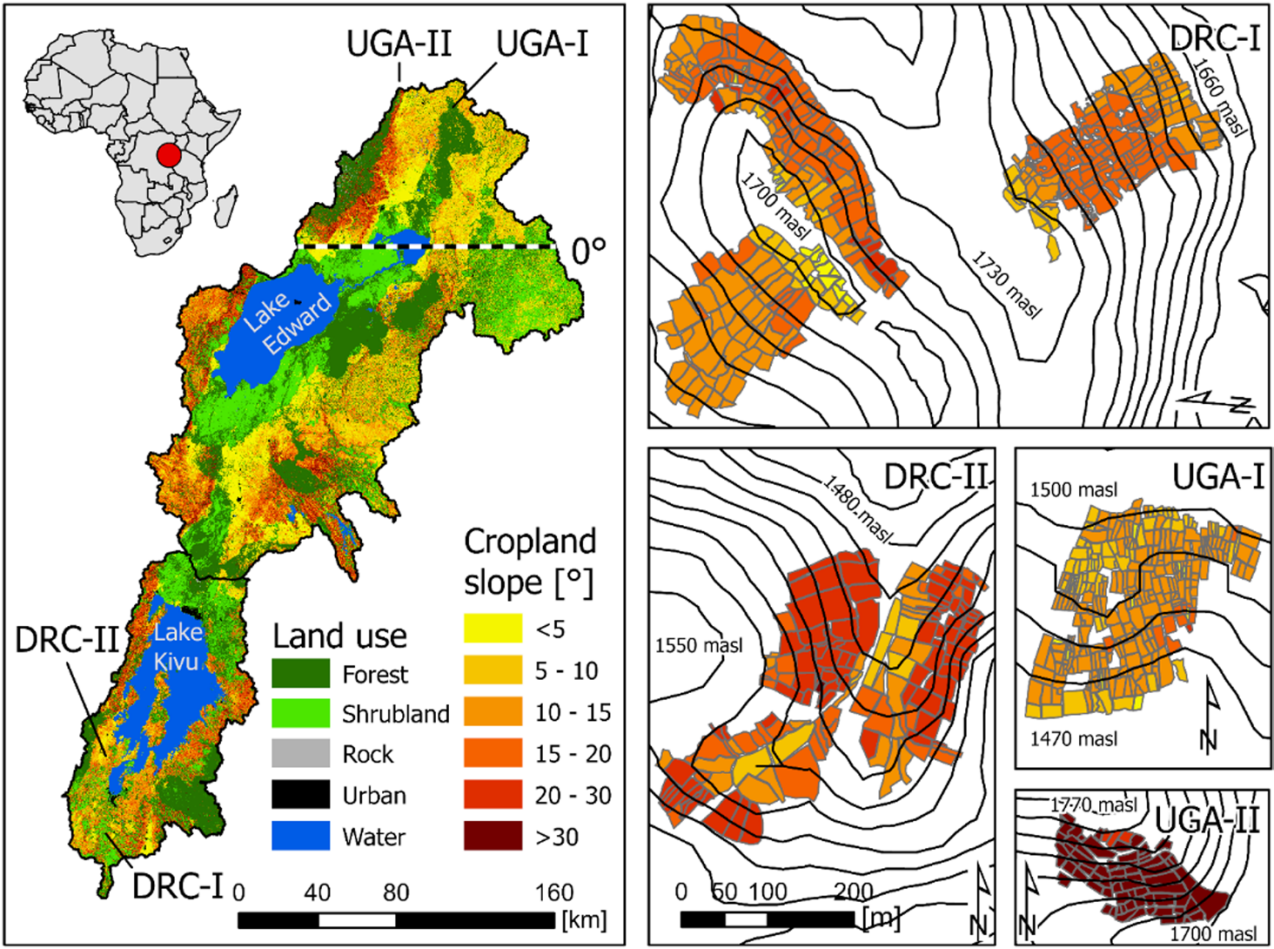
Study area, locations of forest and cropland sites, topography and land use in the White Nile-Congo rift region (land-use data are based on ESA Climate Change Initiative – Land Cover project 2017; map created using ArcGIS Pro 3.0.2 https://www.esri.com/en-us/arcgis/products/arcgis-pro).

To combat food insecurity, a New Green Revolution for Africa^8,9^ based on smart agricultural intensification^7,10^ is initiated by a diverse consortium^9^ e.g. African governments, the Group of Eight G8, philanthropic foundations to close the yield gap and reduce the environmental impact of food production^11^. Smart intensification aims to increase yields by implementing efficient and ecosystem-adapted sustainable farming methods on agricultural land with a high potential for productivity improvements and soil degradation resilience^7^. An area that urgently calls for smart intensification is the White Nile-Congo ridge (NiCo; Fig. 1) region due to rapid population growth (population of Rwanda, Uganda and Democratic Republic of Congo 2020: 150 million – predicted 2100: 430 million; WPR^12^ and thereby substantially rising food demands^13^.

Like many other regions in tropical Africa, the NiCo region faces heavy soil degradation by erosion on arable land, which steadily decreases yield potentials and challenges the sustainable use of soil resources^14–16^. Soil erosion on arable land in the NiCo region is especially governed by a high seasonality and strong erosivity of rainfall^17^ and steep terrain (arable area with slope > 5° = 71%, based on ESA land cover and SRTM DEM, see Fig. 1). Therefore, cropland in the region regularly develops dense networks of rill erosion with high sediment yields^15^. Paired with strong nutrient losses from these deeply weathered soils, accelerated erosion is, therefore, a major threat to the livelihoods of rural communities^18^.

Most of the land is cultivated by smallholder farmers who live in direct proximity to their managed cropland. Smallholder farming systems are characterised by small field sizes (< 0.1 ha) and high land cover dynamics. This leads to highly patchy arable landscapes, which affects the hydrological and sedimentological connectivity of agricultural catchments^19–22^. Agricultural intensification and mechanisation in patchy landscapes with small field sizes typically call for land consolidation^10^ and reducing land management fragmentation. Increasing field sizes causes a higher hydro-sedimentological connectivity^19^ as long as no soil conservation practices, such as terraces or filter strips, are implemented^23^. However, rapid changes in land management and land connectivity from smallholder to intensified farming can have a substantial impact on soil redistribution dynamics by altering soil cover patchiness and corresponding changes in sediment trapping characteristics.

Uncertainties associated with agricultural intensification are partly attributed to the limited understanding of landscape-scale soil erosion dynamics in smallholder farming systems in wet-tropical Africa. The majority of soil erosion studies in wet-tropical Africa either utilise large-scale modelling approaches e.g. Karamage et al.^14^, Nyesheja et al.^24^, Eisenberg and Muvundja^25^, outlet monitoring of runoff plots e.g. Kagabo et al.^23^, Angima et al.^26^, Lewis and Nyamulinda^27^ or field-based observations of rill and gully measurements^15^. None of these approaches fully addresses the issues of soil or rill erosion interconnectivity between fields in agricultural landscapes. Similarly, large-scale models with coarse spatiotemporal resolution do not represent patchy landscapes with dynamic land cover, whereas plot studies only account for erosion processes and neglect depositional processes. Rates and soil redistribution patterns can be assessed using fallout radionuclides such as ^239+240^Pu, which was successfully applied in the NiCo region by Wilken et al.^28^. However, fallout radionuclide tracer assessments integrate all redistribution processes since the 1960s and do not provide information about the frequency and magnitude of erosion events, which limits the insight into event-driven processes. Unmanned aerial vehicles (UAV) can monitor linear erosion features such as rills and gullies and provide process-based insights on field conditions, such as soil cover^29,30^. This is possible since UAVs are efficient tools to carry out aerial imagery that allows user-defined spatial and temporal resolution with low infrastructural requirements (e.g. independent power supply by batteries, no cleared and levelled starting and landing strips needed for copter and vertical take-off and landing UAVs), which is particularly beneficial for studies in remote and rural areas such as smallholder cropland sites in the NiCo region.

This study aims to understand the role of smallholder farming and associated landscape patchiness on rill erosion dynamics. It is hypothesised that rill erosion dynamics in the patchy agricultural landscapes of the NiCo region are controlled by the structural connectivity between small arable fields rather than by slope steepness, and that rill erosion is a regular process occurring throughout the entire cropping season. The specific aims of the study are (i) to quantify the frequency and characterise the inter-field rill erosion network and (ii) to understand the role of smallholder farming systems on rill erosion dynamics.

## Methods

### Study sites and sampling design

**Fig. 2:**
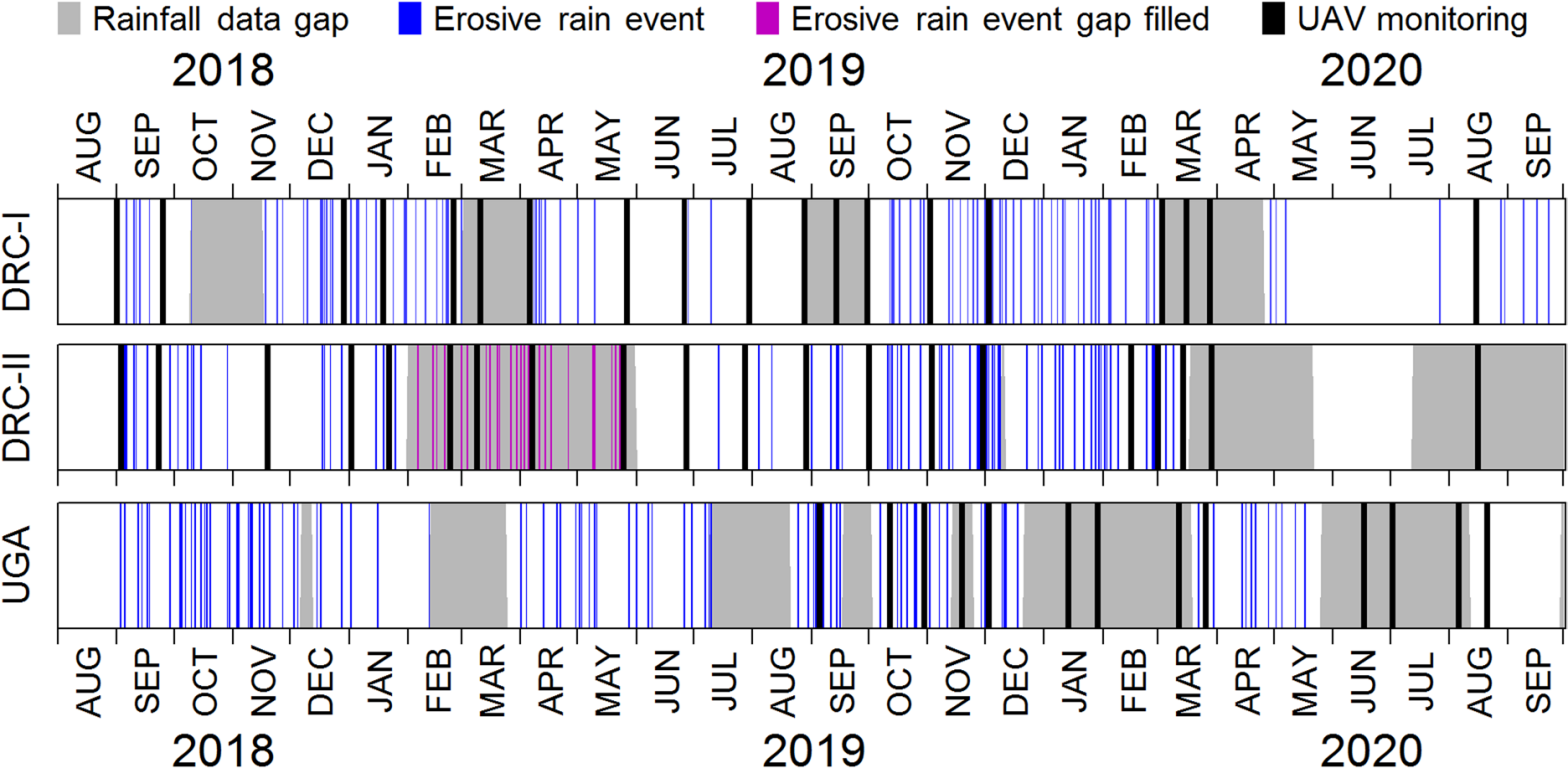
Timeline of UAV monitoring (black bars) and corresponding erosive rainfall events at the study sites. The data gap at rainfall station II was filled by station III for the period from Feb. 01, 2019 to May 31, 2019. Both study sites in Uganda (UGA-I and UGA-II) were UAV monitored at the same dates, but erosive rainfall events were only monitored at UGA-I (see Fig. 1). Number of monitored erosive rainfall events DRC-I: 106, DRC-II: 95; UGA-I: 99.

The White Nile-Congo ridge (NiCo) region extends across the equator and is part of the East African Rift Valley system, specifically the Albertine Rift system (Fig. 1). The NiCo region covers the Lake Edward and Lake Kivu catchments, which are headwater catchments of the White Nile (northern part) and the Congo River (southern part), respectively. The terrain is steep, with an average slope of 10.8° (± SD 9.8°) for the entire study area and 11.4° (± SD 9.0°) for cropland only. The rainfall patterns are driven by two monsoonal rainy seasons (March-May and September-November) and two corresponding dry seasons (December-February and June-August). Rainfall erosivity is high in the rainy season due to frequent storm events Fig. 2, about 20 events per rainy season^31^, each exceeding 10 mm within 30 min, with high rainfall depth and kinetic energy^17^. The NiCo region is a typical example of a smallholder low-input farming system. Mechanised farming in the region is virtually absent and all management operations are carried out by hand without equipment such as tractors or animals. Soil cover conditions in the NiCo region are very patchy and can alternate between bare soil and full vegetation cover in direct spatial proximity^15,28^ (Fig. 1 and S1) due to the small field sizes and differences in individual smallholder field management, such as fallow frequencies, soil tillage, sowing and harvest dates.

Within the NiCo region, four cropland study sites (see Table 1; Fig. 1) were monitored in DR Congo (DRC-I and DRC-II) and Uganda (UGA-I and UGA-II) over two years and one year, respectively. The study sites and flight plans were selected to represent a range of cropland characteristics in the NiCo-region in terms of topography, main crops, field architecture and topsoil properties. Moderately steep slopes (< 20°) are represented in the study sites DRC-I and UGA-I, while DRC-II (20° − 30°) and UGA-II (> 30°) represent steep slopes (see Fig. 1). Soils in the NiCo region are predominantly strongly weathered Nitisols and Acrisols^32^. However, due to the tectonic activity in the region, periodic replenishment of soils with rock-derived nutrients from pyroclastic deposits occurs to varying degrees at local scales^33–35^. Pyroclastic deposits can be found in the Ugandan study sites (UGA-I and UGA-II) and lead to a somewhat lower topsoil clay content (DRC: 65%, UGA: 35%)^32^ and consequently higher soil erodibility compared to the DR Congo sites (DRC-I and DRC-II). Another major difference between the study sites is the hydro-sedimentological connectivity. In DRC-I and DRC-II, a network of ditches along the field borders routes runoff rapidly off the slopes and, furthermore, the main field orientation is in the direction of the hillslope. In UGA-I, the fields are rectangularly shaped, while at the steep slope of UGA-II the field orientation is contour parallel. Both study sites in Uganda do not have a network of ditches along the field borders, while in UGA-II, stabilising grass strips are cultivated at the downslope field borders. In general, the mean field sizes in DR Congo are larger (289 m²) compared to Uganda (116 m^2^). The main crop differs between the northern (Uganda) and southern (DR Congo) parts of the NiCo region. While in DR Congo Cassava (*Manihot esculenta*) is predominantly grown, in Uganda, sweet potato (*Ipomoea batatas*) and groundnut (*Arachis hypogaea*) are the main crops. In both study sites, corn (*Zea mays*) is regularly cultivated.

**Table 1:** Number of monitoring times, flight plans, fields and field sizes and covered area of different study sites.

	DRC-I	DRC-II	UGA-I	UGA-II
*n*-flights	20	19	13	14
n-flight plans	4	3	2	1
Area [ha]	7.8	5.6	3.3	1.0
Mean field size [m^2^]	251	368	109	144
Fields	311	152	298	72

### UAV monitoring

A consumer-grade UAV platform (Phantom 3, DJI, China) was used to operate a high accuracy (spatial error < 10 cm) georeferencing system (Reach M+, Emlid, China) based on PPK-GNSS (post processing kinematics-global navigation satellite system) technology to locate RGB images (Hero3 black, GoPro, USA). For more details regarding the UAV setup see Zhang et al.^36^. To achieve an accurate multi-temporal image overlay, the stationary base station (Reach RS+, Emlid, China) was positioned above concrete foundations of 1 m x 1 m x 0.5 m, built at each study site in DR Congo and Uganda. UAV monitoring was scheduled at fixed intervals, with monitoring during the rainy seasons carried out on a two-week frequency. During the dry seasons, monitoring was conducted at a four-week frequency, with some irregularities due to technical issues and the COVID-19 crisis (Fig. 2). Monitoring continued during the dry season because rare rainfall events still occur in the study region during this period. Monitoring started in the DR Congo in September 2018 and in Uganda in November 2019 and ended for both areas in September 2020. At the DR Congo and Ugandan sites, seven and two flight plans were regularly carried out, respectively (Fig. 1; Table 1). Over all areas, the monitoring covered 833 individual fields (DRC-I: 311, DRC-II: 152, UGA-I: 298, UGA-II: 72) over a total area of 18 ha. The monitoring design aimed to achieve a landscape-scale assessment that provided sufficiently high image resolution for rill detection and simultaneously large spatial coverage. Therefore, flight plans at an approximate altitude of 30 m with 80% frontal and 60% side overlap were set up for automated image acquisition. The multiple images were stitched into orthomosaics for each flight plan (Pix4D mapper v4.5.6, Pix4D, Switzerland) of an average ground sampling distance of 3.7 ± 0.5 cm.

### Rainfall data

Meteorological monitoring data from Doetterl et al.^31^ were used to understand general rainfall characteristics and, in particular, relative erosivity variations of individual rainfall events in the study region. Therefore, the frequency and magnitude of erosive rainfall events were assessed using high-resolution tipping bucket rainfall monitoring data (5 min interval; ATMOS 41, Meter, Germany), carried out at four locations in the study region. DRC-I and UGA-I were equipped with individual rainfall stations in direct proximity to the monitored fields that were less than 1 km apart. The closest stations to DRC-II are in 13 km distance, located at the city of Bukavu and at the Kahuzi Biega National Park (province of South-Kivu, DR Congo). For UGA-II, no representative rainfall data is available as the closest station is 22 km apart and UGA-II is located at the slopes of the Rwenzori mountains (Western province, Uganda), which cause hydrometeorological conditions (strong orographic rise) that are not comparable to UGA-I. Rainfall erosivity was calculated on an event basis (min. 10 mm total or > 5 mm of 30 min rainfall sum, events are separated by a 6 h rainfall gap). The rainfall kinetic energy was derived using the KE-I relation according to Brown and Foster^37^ and subsequently multiplied by the 30-minute maximum rainfall intensity (mm h^− 1^). The KE-I relation of Brown and Foster^37^ has been positively evaluated in the study region by Bagalwa et al.^17^. In the case of technical issues, the erosivity data for DRC-I were gap-filled using data from Bukavu and Bugulumiza, located at distances of 16 and 30 km, respectively.

### Erosion classification

The field borders were visually delineated based on the initial orthophoto of the monitoring at each study site. Subsequently, the fields were classified into five classes representing the erosion potential (class i and ii) and rill erosion intensity (class iii, iv and v): (i) fields with limited rill erosion susceptibility due to high soil cover > 60%, (ii) erosion prone fields with moderate to low soil cover < 60% without signs of rill erosion, (iii) fields with a single rill or very limited spatial extent of rill erosion i.e. area affected is smaller than 10% of the field, (iv) strongly eroded fields with multiple erosion rills (spatial extent 10–50%) and (v) fields with signs of rill erosion that form a dense rill network spatially expanding over the majority of the field (> 50%). To track rill development, the first time a rill is observed is provided as additional information in the classification.

Classification of erosion features visible in the imagery data was carried out through manual inspection of all data by two experts, the authors Haist (expert 1) and Wilken (expert 2). An initial classification was conducted by expert 1 for the entire dataset. Subsequently, expert 2, with extensive fieldwork experience in the study region, evaluated and corrected the entire classification, with particular focus on fields with low soil cover or detected rill erosion. Expert 2 investigated each field with classified rill erosion individually using the raw images (before orthomosaic stitching), allowing for different viewing angles at the highest possible resolution. Conflicts between the two classifications were assessed using a confusion matrix that unravels class-specific classification uncertainties. Furthermore, the mean balanced accuracy and F1 score were calculated to address the class-specific and total uncertainty of the classification.

### Analysis of field conditions

To assess the controls whether fields develop rill erosion or not, a subset of fields (182 fields with rill erosion and 534 low soil cover fields without rill erosion) were analysed with respect to the following conditions: (i) slope steepness, (ii) vegetation cover and (iii) soil conditions. Field-based slope steepness was derived from structure-from-motion DEMs (digital elevation models), which were generated from images captured during the monitoring flights (see section “UAV monitoring” and Fig. 1). The vegetation cover was visually classified into 5 classes: <10%, 10–20%, 20–40%, 40–60%, and > 60%. The soil condition was categorised into four classes: settled bare soil without signs of recent tillage operation, mounds for sweet potato plantation, plant stubbles with intact roots and visible rills under fully developed vegetation cover. Ordinal and multinomial logistic regression analysis were used to assess the relationship between slope degree, slope length factor (USLE L-factor^38^, soil cover, and soil condition on rill erosion severity of individual fields at specific monitoring times. This method was chosen due to its ability to handle categorical dependent variables. All data analyses were conducted using R version 4.4.3 (R Core Team, 2025).

## Results and discussion

### Evaluation and limitations

With a 3D positional accuracy of approximately 10 cm, the UAV setup used in this study enables precise image georeferencing and topographic data processing^36^. However, a 10 cm spatial error is still not sufficiently accurate for a multi-temporal DEM change detection analysis, as the mean rill depth is 12.5 cm based on field observations carried out by Heri-Kaze and Bielders^15^ is close to the range of the location error. Using permanent structures (e.g. house roofs or rocks) as reference points would push the georeferencing error to a minimum at the cm-level. However, alterations in vegetation cover and soil surface dynamics, such as elevation of the soil surface caused by a reduction in soil bulk density and changes in roughness due to soil tillage, limit direct comparison of multi-temporal digital elevation models^39^(DEM). Therefore, visual identification and classification of rill erosion were carried out within this study. Errors in the classification of erosional features were assessed with a confusion matrix that illustrates class-specific conflicts between the classifiers (Table 2). In general, the evaluation between the two classifications of expert 1 and 2 showed a high agreement (Table 2) with a mean balanced accuracy and F1 score of 0.97 (class min.: 0.95) and 0.93 (class min.: 0.87), respectively. Classes with signs of rill erosion identified by expert 1 were not confirmed by expert 2 in 38 fields (88% agreement regarding the presence of rills), considering all monitoring dates at all sites (11033 fields). In 13 individual fields, expert 2 identified rills not detected by expert 1. A conflict between the rill-intensity classification was found in 4.8% of fields with signs of rill erosion. These results indicate that a careful visual classification and evaluation of high-resolution aerial imagery carried out by multiple experts is a reliable approach to assessing rill erosion dynamics in smallholder farming systems with high spatiotemporal variations.

**Table 2:**
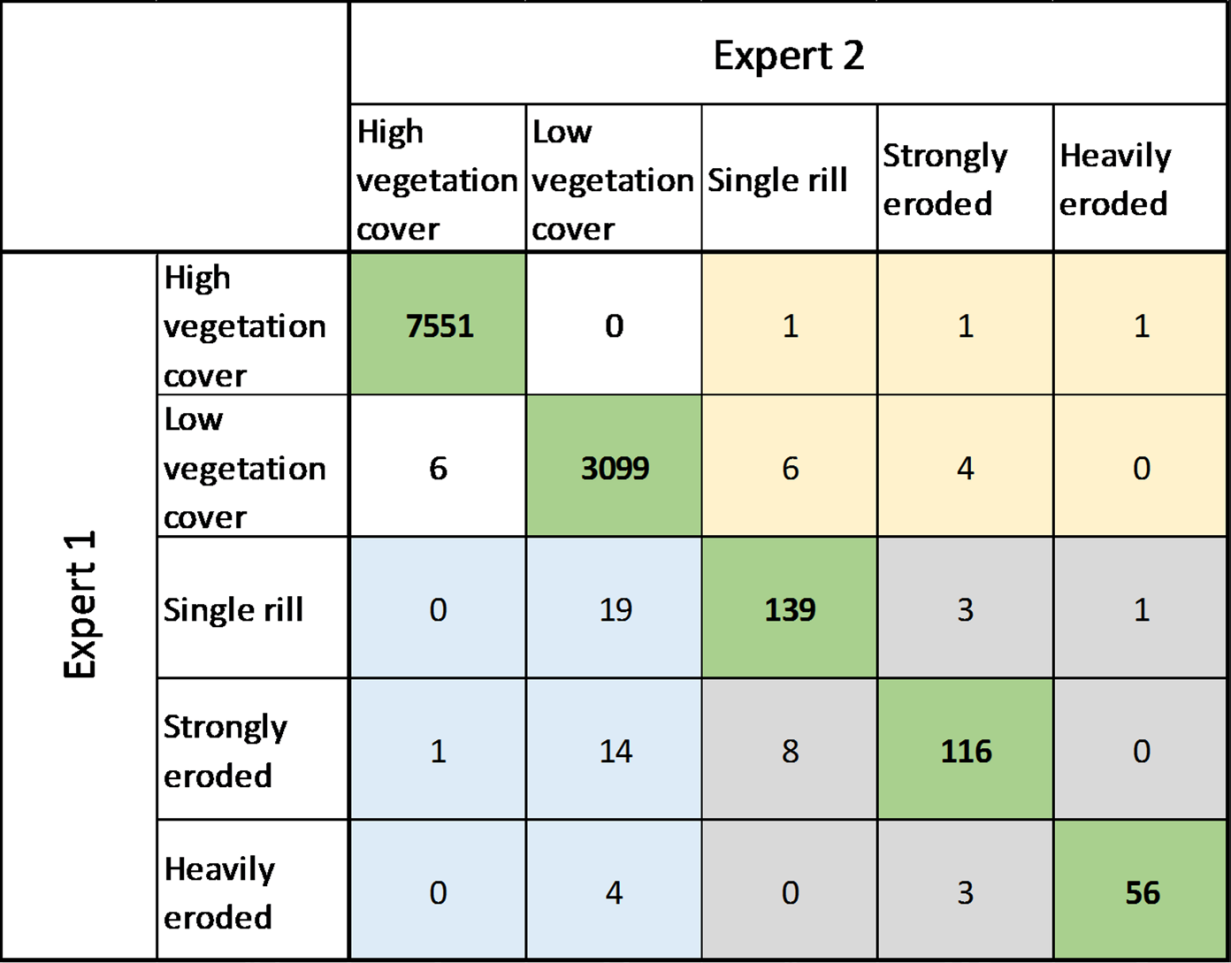
Confusion matrix of field-based rill classification.

Bold and green cells mark fields without disagreement between the two classifications. Yellow cells identify fields with non-identified rills by expert 1 (“false” negative), while blue cells highlight fields where expert 1 identified fields but expert 2 disagreed (“false” positive). For the grey cells both experts identified rills but disagreed on the rill intensity.

The visual classification restricts detection to linear erosion features such as rills and ephemeral gullies. Diffuse processes such as sheet erosion cannot be identified using this method. Moreover, visual mapping is time‑consuming and therefore difficult to scale to larger areas or broader regional assessments. Practical and logistical constraints restricted the temporal sampling frequency, with the highest achievable resolution being a two‑week interval. However, monitoring rill erosion at the scale of individual events would require daily image acquisitions, given the frequent occurrence of multiple storm events within short periods. Consequently, the monitoring does not capture individual storm events but rather the dynamics of rill‑erosion event series.

### Frequency of rill erosion

**Fig. 3:**
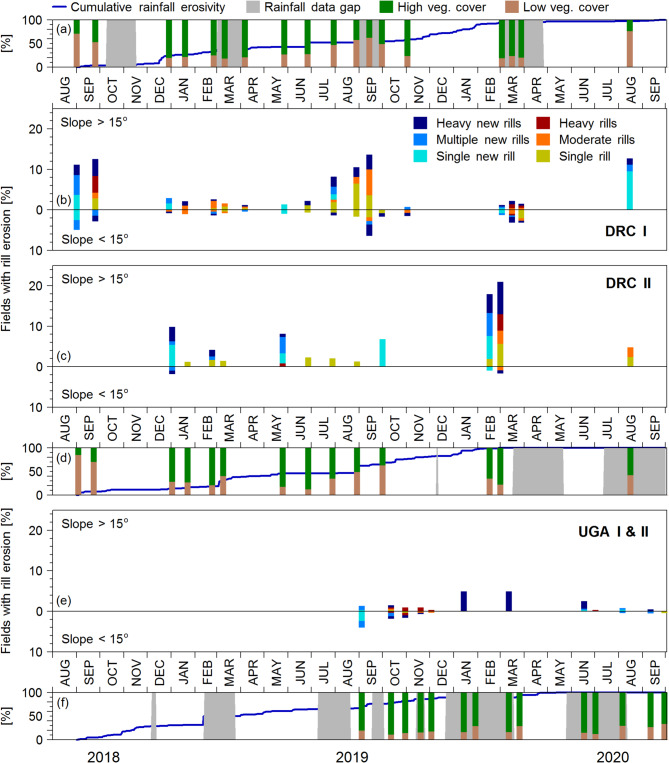
Proportion of low vegetation cover (< 60%, brown) versus high vegetation cover fields (> 60%, green) and cumulative rainfall erosivity (panels a, d and f). Relative number of low vegetation cover fields affected by rill erosion (panels b, c and e). Blue colours indicate rills that have been newly developed since the last monitoring date, while yellow-red colours indicate rills that have already been observed at the previous monitoring date.

**Fig. 4:**
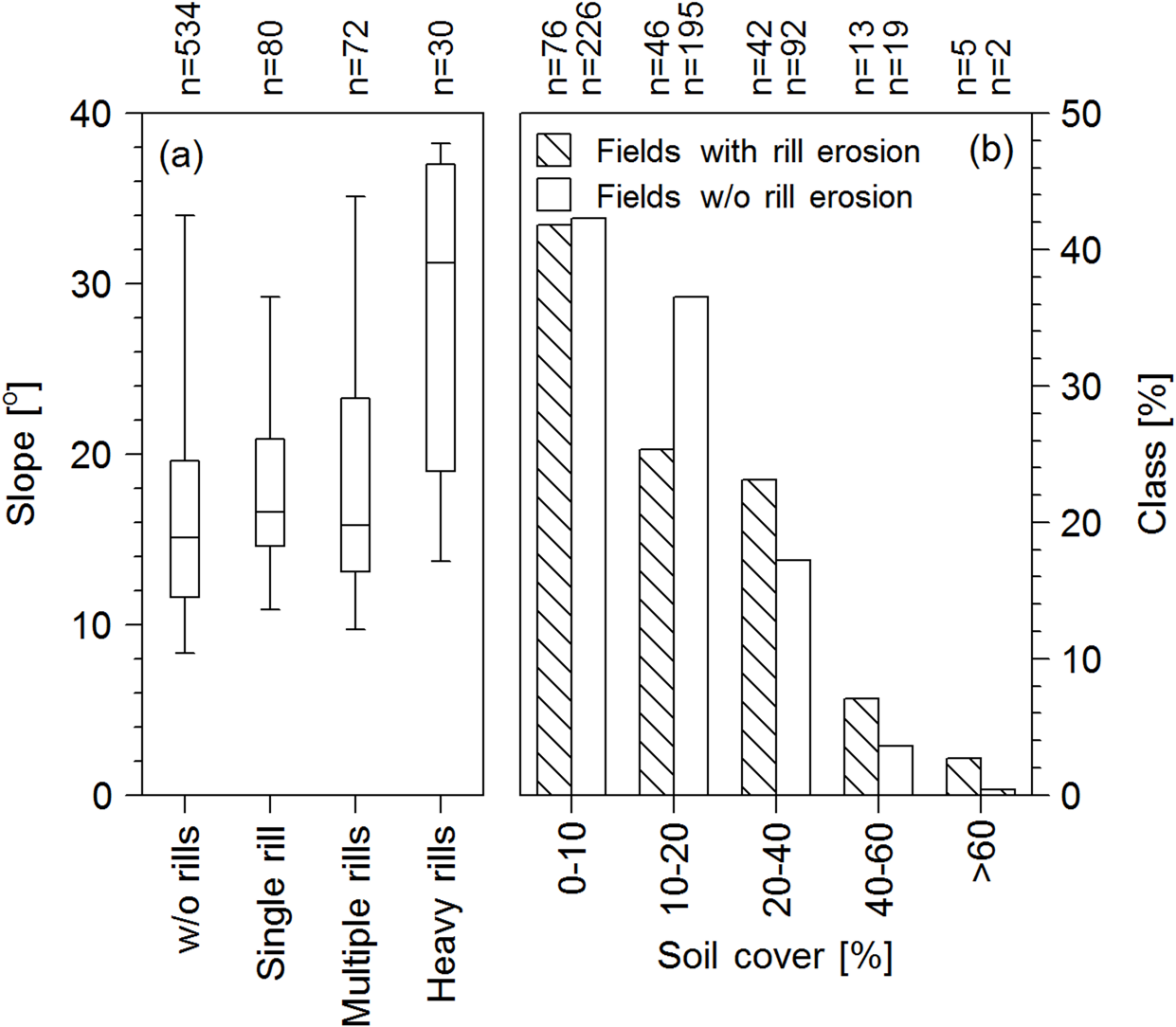
Properties of fields with rills and without rills. (a) Boxplot of fields with rill erosion and without (w/o) rill erosion reflected against slope. (b) Comparison between fields with and w/o rill erosion against soil cover conditions.

The data shows that cropland rill erosion in the NiCo region is taking place regularly (Fig. 3). This is caused by a high frequency of erosive rainfall events that occur roughly every 4 days during rainy season (about 20 events within 3 months; Fig. 2). As a result, fields with recent rill development were identified for the majority of the monitoring dates (newly developed rills/total monitoring dates: DRC-I 17/18; DRC-II 6/14; UGA I & II 9/14). However, it was observed that rill formation is highly localised and does not incise across multiple fields. Often, fields under visually similar conditions do not develop rills on the same monitoring date and the same impact of erosive rainfall. Hence, only a small fraction of fields developed new rills between monitoring dates where erosive rainfall took place (DRC-I: 5.6%; DRC-II: 15.4%; UGA-I and II: 9.8%; Fig. 3). The ordinal logistic regression analysis showed that the effects of soil cover, soil structure, and the slope length factor were negligible, as indicated by z-values between − 1.3 and 1.3, indicating very limited influence on the probability of rill erosion severity. Slope is the only factor substantially impacting the likelihood of rill development. Especially the probability of heavy rill erosion is positively correlated with slope steepness, which is illustrated by Fig. 4 and supported by the multinominal logistic regression analysis (z value: 5.1). However, very steep slopes (> 30°, *n* = 71) are almost exclusively located at the study site UGA-II (Fig. 1). Hence, rill erosion development is not just simply triggered by erosive rainfall meeting low soil cover conditions on a steep slope or long erosive slope length. Another potential control is the time since the last tillage operation, which is a relevant parameter for soil erodibility and soil susceptibility to rill erosion as observed in the mid-latitudes^40^ due to a weakened soil structure that benefits soil detachment^41^. Unfortunately, the visual identification of tilling actions, even from high-resolution and high-recurrence aerial imagery, was hardly possible due to timely post-tillage soil surface smoothening by a high rainfall frequency. Beyond the factors already mentioned, rill erosion can be influenced by a range of variables, including microtopography, root density and structure of different crops, crop types and rotation, soil structure, soil fauna and antecedent soil moisture. The interactions and roles of these mechanisms call for further research, including the integration of ground-based field and soil condition monitoring. Nevertheless, the results of this study suggest that model-based soil erosion predictions for regions under smallholder farming may call for parameterisations that go beyond standard paradigms and assumptions associated with the occurrence of erosional events and the features they form along hillslopes.

**Fig. 5:**
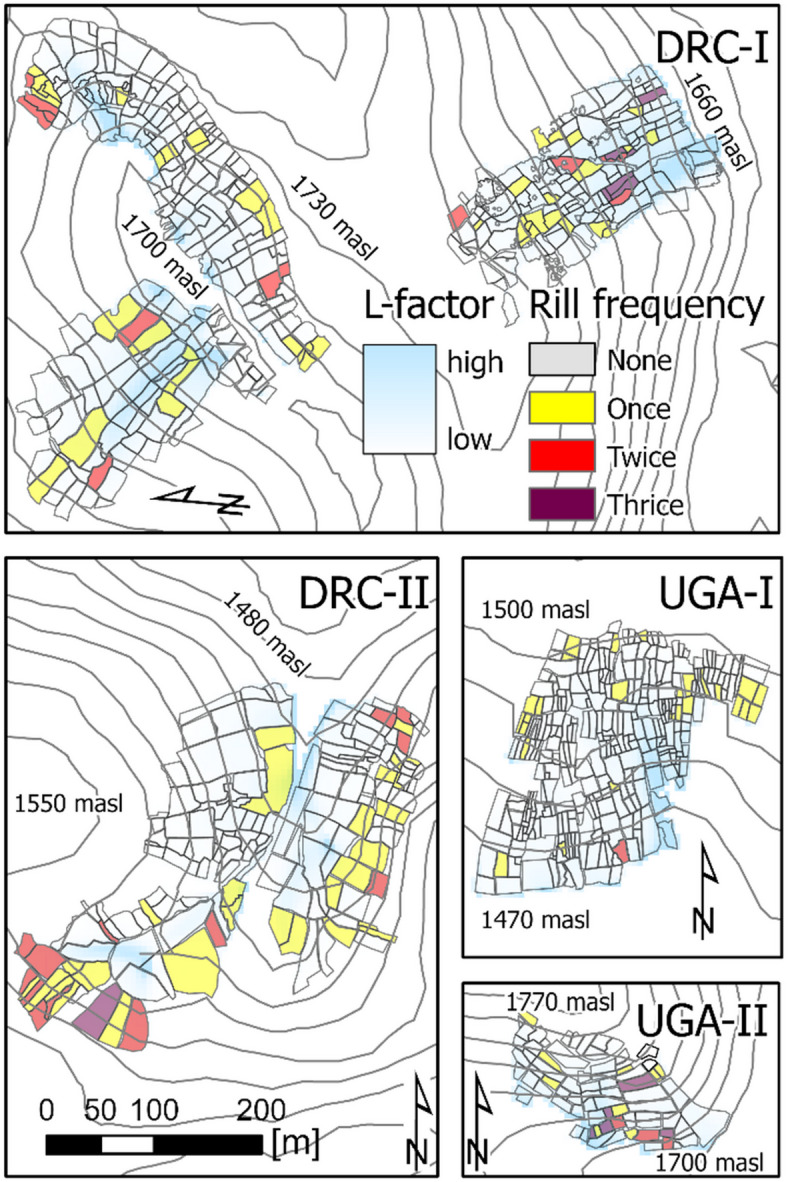
Rill erosion frequency during the monitoring and USLE L-factor giving the erosive upslope length. Fields with newly developed rills were counted that were separated by at least one monitoring time without rills. Map created using ArcGIS Pro 3.0.2 https://www.esri.com/en-us/arcgis/products/arcgis-pro.

The rill frequency in individual fields (the number of times newly developed rills were observed at each individual field) was assessed to detect potential spatial patterns or spatial distribution along the slope in rill erosion dynamics (Fig. 5). Of 833 monitored fields, 18% developed erosion rills over the course of monitoring. Only a few clusters of fields that developed rills multiple times were found (Fig. 5). Most of those fields only developed rills a single time during the monitoring period (72%), while 28% of the fields developed rills multiple times. One-third of the monitored fields in DR Congo developed erosion rills multiple times (DRC-I 30%; DRC-II 33%). The contrast between the Ugandan sites is more pronounced, with 44% and 6.9% of fields developing rills multiple times during the monitoring period in UGA-I and UGA-II, respectively. Hence, in UGA-II rill erosion is somewhat localised to specific fields due to extremely steep slopes (> 30°) and high runoff concentrations. However, at all other study sites, rill development is not confined to specific fields. Therefore, rill erosion in the study region is neither limited to nor focused on particular fields or slope positions, but occurs mainly without a distinct spatial distribution, making event-based prediction of rill erosion hardly possible.

### Smallholder farming and soil redistribution dynamics

Smallholder farming in DR Congo and Uganda exhibits clear differences in management and hydrological and sedimentological conditions. In the DR Congo, the general field size is about double that of Uganda, and the longest field dimension is oriented downslope. As a result, the erosion susceptibility due to the general field architecture in Uganda is lower compared to fields in DR Congo, leading to fewer fields with rill erosion (see Fig. 3b, c, e). The large impact of these differences in field architecture and drainage between the sites in DR Congo and Uganda is supported by strongly depleted ^239+240^Pu inventories in DRC-I versus UGA-I, with a strong contrast between severe erosion and large deposition observed within short distances^28^. Smallholder farming in the NiCo region resulted in many small fields (DRC: 35 fields ha^− 1^; UGA: 86 fields ha^− 1^) and farmers with individual management practices, leading to a patchy landscape with highly variable soil cover. The differences in management practices, as well as the complex hydrological connectivity between fields, lead to high spatial variability in patterns and signs of erosional soil translocation. For all sites and monitoring dates, low soil cover erosion-susceptible and high soil cover erosion-protected fields were simultaneously present during both the dry and wet seasons (see Fig. 3a, d, f). During the rainy and main cropping seasons, the percentage of fields with soil cover below 60% was as follows: DRC-I at 36%, DRC-II at 49%, UGA-I at 17%, and UGA-II at 18%. Landcover patchiness with pronounced management differences between connected fields effectively reduces slope length for runoff concentration and, correspondingly, rill erosion. This is illustrated by the lack of statistical relationships between rill erosion severity and the slope length factor in the multinomial linear regression analysis. Furthermore, Fig. 5 supports this finding as only a few neighbouring fields develop rills multiple times. Furthermore, a drainage network along the field borders characterises the DR Congo study region^42^ that routes water off arable fields down to the valley bottoms (see Fig. S1). Hence, the drainage network decreases inter-field connectivity while simultaneously increasing sediment delivery to the river network. Arable fields in Uganda do not have such intentionally built sediment transport pathways that benefit intra-slope deposition. These patterns do not necessarily follow slope steepness or length due to the complex inter-field connectivity in smallholder farming systems, which can lead to either erosion or deposition at the field scale. This soil redistribution dynamics leads to high intra-slope variability in soil properties such as soil organic carbon stocks, as demonstrated in a study by Reichenbach et al.^43^ conducted in the NiCo region. The latter study demonstrates that smallholder farming practices and land use patchiness control soil redistribution dynamics in the study region. Smallholder farming plays a crucial role in disrupting hydrological and sedimentological connectivity between fields, a factor that is not adequately represented in current model-based estimates. However, the processes and drivers for such an implementation are not fully understood. Plot and field experiments under natural rainfall in wet tropical Africa have shown substantial soil erosion rates under bare soil conditions (e.g. maximum annual soil loss rates in plot experiments, Angima et al.^26^: 502 Mg ha^− 1^ yr^− 1^, slope 22°; Byers et al.^44^: 859 Mg ha^− 1^ yr^− 1^, slope 28°; Lewis and Nyamulinda^27^: 190 Mg ha^− 1^ yr^− 1^, slope: 31°). On a landscape scale, modelling studies (USLE-based) predict a large range of long-term average soil loss rates for different catchments (Byizigiro et al.^45^, 302 km^2^: 38 Mg ha^− 1^ yr^− 1^; Chuma et al.^42^, 257 km^2^: 24 Mg ha^− 1^ yr^− 1^; Eisenberg and Muvundja^25^, 120 km^2^: 48 Mg ha^− 1^ yr^− 1^; Karamage et al. ^46^, 8350 km^2^: 490 Mg ha^− 1^ yr^− 1^; Kulimushi et al.^47^, 57684 km^2^: 138 Mg ha^− 1^ yr^− 1^; Lufafa et al.^48^, 30 km^2^: ~33 Mg ha^− 1^ yr^− 1^; all listed studies are located in the NiCo region of eastern DR Congo and Rwanda). However, plot experiments assess potential soil loss without accounting for deposition. Large-scale modelling approaches, which are mostly USLE-based, do not account for smallholder-induced land-cover patchiness and corresponding effects on the hydrological and sedimentological connectivity. Therefore, large-scale erosion modelling of regions dominated by smallholder farming necessarily overestimates soil loss, as the slope length calculation (USLE L-factor) is typically based on moderate resolution DEM data (e.g. SRTM 30 m x 30 m), which cannot address small hydrological features (e.g. ditch network in DRC) and ignores the high land cover variation in the study region and thereby the in-field deposition of mobilised soil material. The long-term (~ 55 yrs.) effect of smallholder-driven landcover patchiness in tropical cropland on inter-slope soil redistribution variability was demonstrated by Wilken et al.^28^ using ^239+240^Pu fallout radionuclide tracers at the study sites DRC-I and UGA-I. The highest soil loss rates found by Wilken et al.^28^ were 51.4 Mg ha^− 1^ yr^− 1^ at study site DRC-I. Heri-Kaze and Bielders^15^ carried out rill erosion monitoring of individual fields near the DR Congo study sites over two years (i.e. four rainy seasons) and found soil-loss rates ranging from 66 to 140 Mg ha^− 1^ yr^− 1^. The high erosion rates in steep arable land observed by Wilken et al.^28^ and Heri-Kazi and Bielders^15^ are in the same range as, or lower than, the rates estimated from plot and large-scale modelling studies in the region, but substantially lower than some of the more extreme estimates referenced above. While still high compared to erosion rates in temperate climates, the estimates indicate that landscape patchiness created by smallholder farmers can contribute greatly to reducing soil erosion losses in the region. Of concern is that agricultural optimisation through modernisation and intensification to increase yields in tropical Africa^1,7,8^ typically calls for land consolidation^49^ for the efficient use of farming machines. In the study area, this might result in an extreme acceleration of soil erosion rates due to increasing slope lengths and homogeneous bare soil conditions following tillage and harvest operations across larger areas. Without massive investments in soil protection and extensive fertiliser applications to replace nutrients lost from eroded topsoil, large yield declines are likely to follow. There is an urgent need to increase agricultural productivity that calls for modernisation and intensification in the study area. Even though the discussed implications extend beyond the scope of the measured data and should be interpreted as broader contextual considerations, it remains essential to acknowledge that intensification calls for alignment with location-specific sustainable land management practices without causing extreme degradation. Furthermore, agricultural intensification can also offer an opportunity to sustain soil ecosystem services, as higher cropland productivity may free up land that could be converted to grassland or reforestation in erosion-prone areas.

## Conclusions

In this study, rill erosion monitoring in smallholder farming systems of wet tropical Africa was carried out at more than 800 fields over two years. A visual classification of UAV-based orthophotos demonstrated that rill erosion occurs frequently throughout the entire monitoring period. The occurrence of rill erosion has therefore proven to be highly selective, as not all low vegetation cover fields showed rill formation after erosive rainfall events, even though the field conditions appear similar. Extreme land cover patchiness due to small fields with individual management leads to complex hydrological and sedimentological connectivity in the study area. Thereby, rill formation rarely traverses into the downslope-located fields, as variations in field conditions force local deposition. This is a highly relevant but ignored process in large-scale soil erosion modelling, necessarily causing an overestimation of predicted soil erosion rates. Thus, there is a critical need to advance process‑based understanding of structural and functional landscape connectivity patterns and to establish model parameterisations tailored to local soil erosion conditions. This study further underlines the relevance and mitigation potential of smallholder farming systems for soil redistribution processes in the wet tropics of Africa. Therefore, the potential intensification and mechanisation of these agroecosystems need to be critically evaluated, as they may accelerate soil degradation, driven by larger field sizes and higher hydrological and sedimentological connectivity.

## Supplementary Information

Below is the link to the electronic supplementary material.

Supplementary Material 1

## Data Availability

The data of this study are available upon request from the corresponding author.
