# Supplementary material for: Rill erosion dynamics in smallholder farming systems of wet tropical Africa
Source: Sci Rep. 2026 May 21;16:15863. doi: 10.1038/s41598-026-50821-7 (PMC13194724; doi:10.1038/s41598-026-50821-7)
Supplement: Supplementary file 1 — Supplementary Material 1 [file 41598_2026_50821_MOESM1_ESM.docx]

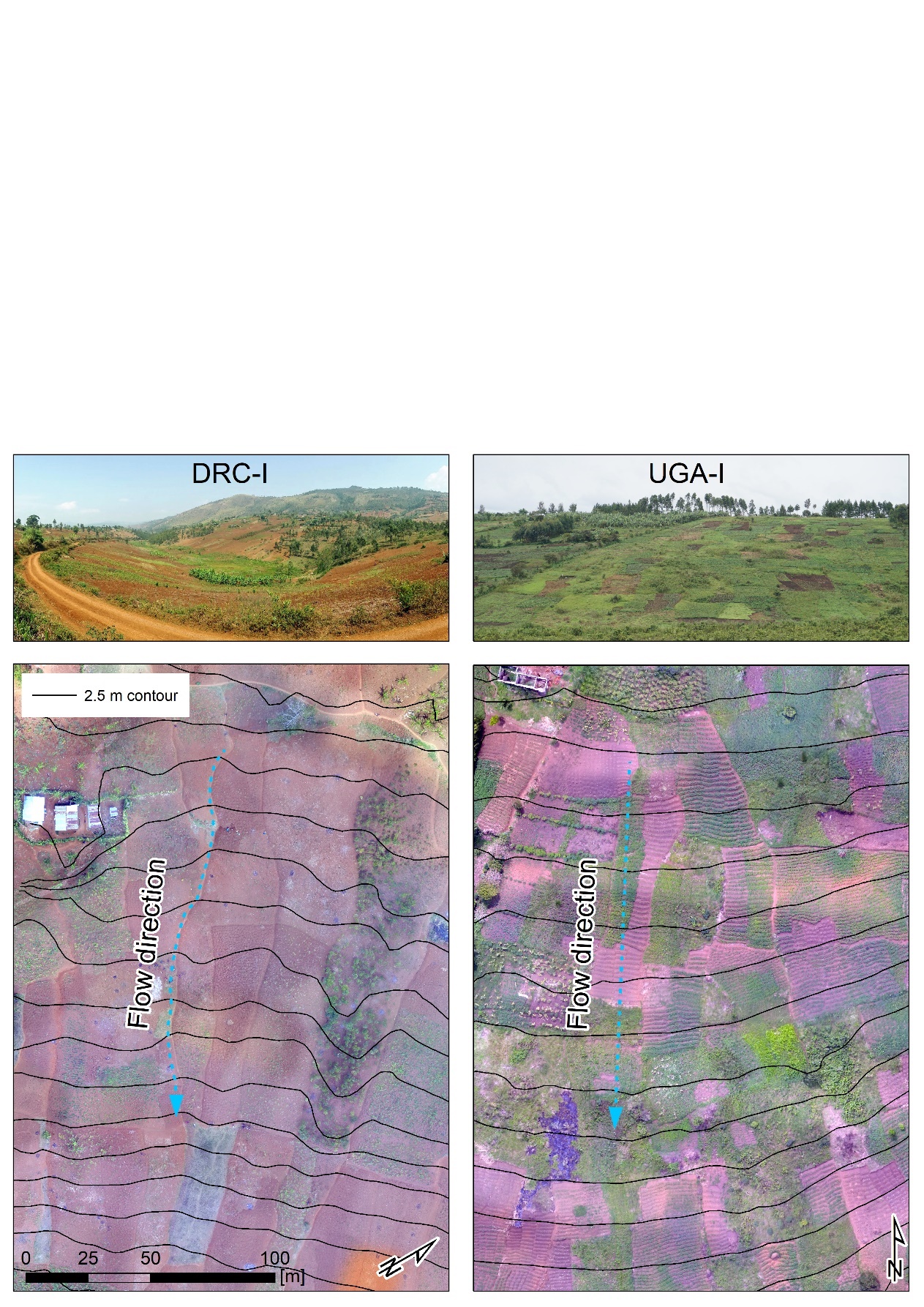


Figure S1: Overview photos (upper panel) and unmanned aerial vehicle based orthophotos (lower panel) of study sites DRC-I and UGA-I. The flow direction arrow in DRC-I (dashed blue arrow in lower left panel) exemplarily highlights the drainage network along the field borders that are characteristic for the study sites in the DR Congo. Map created using ArcGIS Pro 3.0.2 https://www.esri.com/en-us/arcgis/products/arcgis-pro.
